# A stochastic wideband propagation-graph channel model for plants-affected drones air-to-ground mmWave communications

**DOI:** 10.1371/journal.pone.0333929

**Published:** 2025-10-29

**Authors:** Jiachi Zhang, Liu Liu, Shu-bin Li, Yifei Xu

**Affiliations:** 1 School of Police Information, Shandong Police College, Jinan, China; 2 School of Electronic and Information Engineering, Beijing Jiaotong University, Beijing, China; 3 Shandong Engineering Research Center of Intelligent Traffic Control and Guidance Technology for Public Security, Shandong Police College, Jinan, China; 4 School of Management Science and Engineering, Shandong University of Finance and Economics, Jinan, China; Guangdong University of Petrochemical Technology, CHINA

## Abstract

Unmanned aerial vehicles (UAVs) are experiencing extensive worldwide application across various fields, particularly in outdoor scenarios that often involve vegetation. A comprehensive understanding of the air-to-ground (A2G) wireless link channel and fading characteristics is crucial for the deployment and optimization of communication systems. In this paper, we propose a A2G wideband wireless channel model that integrates line-of-sight (LoS), reflection, and scattering propagation mechanisms at typical millimeter-wave (mmWave) bands based on the stochastic propagation-graph model. First, a geometric fractal tree modeling method is introduced to represent a single tree, the size and distribution of trees are modeled based on the stochastic theory. The propagation-graph (PG) model is then employed to simulate the A2G channel impulse response (CIR) at 28 GHz, taking into account maximal propagation delay constraints. On this basis, we investigate the spatial cross-correlation function (CCF) at different positions and effects of different UAV heights and circular movement radii on delay spread. Additionally, we study key small-scale statistical channel properties, including the power delay profile (PDP), power angular profile (PAP), and Doppler power spectrum density (DPSD). Our simulation results demonstrate that vegetation significantly impacts channel dispersion in spatial-temporal-frequency domains, and our model effectively captures the non-stationarity of UAV A2G channels.

## Introduction

The global applications of unmanned aerial vehicles (UAVs) have experienced explosive growth across various industries, revolutionizing traditional technologies and enabling new capabilities due to their broad coverage, high mobility, and flexibility [1]. Benefiting from their small size, lightweight design, and reliance on advanced battery technology [2], UAVs are undergoing rapid deployment expansion in increasingly diverse environments. These applications span logistics and transportation [3], smart agriculture [4], infrastructure inspection, etc. UAV application scenarios, especially those outdoor scenarios including mountainous regions, forested areas [5], agricultural orchards, etc., inevitably involve the presence of vegetation, which can significantly impact the performance of UAV-ground communication systems. As vegetation interacts with electromagnetic waves, it can cause propagation blockage, attenuation, scattering, and multipath effects that degrade the received signal quality and reliability. Therefore, it is crucial to investigate the influence of vegetation on radio wave propagation when designing and optimizing UAV communication systems for these environments. Additionally, millimeter-wave (mmWave) technology, which operates in the 30 GHz to 300 GHz frequency range, plays a crucial role in next-generation mobile systems by supporting higher data rates compared to lower frequencies. Consequently, integrating UAVs with mmWave technology presents an ideal solution for efficiently transmitting large volumes of data [6].

UAV communications consists of air-to-air (A2A) and air-to-ground (A2G) links. A2A links involve communication between flying UAVs, which predominantly experience the line-of-sight (LoS) radio propagation. In contrast, A2G links refer to communication between UAVs and ground-based user terminals, where radio waves can be easily obstructed or affected by low-height obstacles such as trees or buildings, particularly in woods, forest, or urban environments. Understanding the characteristics of UAV A2G wireless channel propagation is crucial for further system design and optimization. Although extensive research has been conducted on radio propagation modeling and the characteristics of mmWave communications for UAVs, e.g., [2,6–8], most researchers has focused on theoretical models and measurement validation, with limited attention to the impact of vegetation. In [9], authors performed real-time measurements and characterization of the A2G channel for UAV communications in a rural area. The resulting measurement campaign provided key insights into channel properties and impairments. Nonetheless, there still has been little research specifically addressing UAV A2G channel modeling and characterization in vegetation environments densely populated with plants or trees. Consequently, a significant research gap exists in this area, which this paper aims to address by providing an in-depth analysis and valuable insights.

The remainder of this paper is organized as follows. In Sect 1, we present related work on channel modeling and characterization, along with the contributions of this study. Sect 2 introduces the fractal geometric tree modeling method. Besides, we detail the stochastic propagation-graph channel model and explain the scatterer selection method based on maximal propagation delay constraints. In Sect 3, we introduce the key channel characteristics, including the delay spread, spatial cross-correlation function (CCF), and power density profiles. Sect 4 presents the channel simulations, discussing the plants-affected mmWave channel fading characteristics. Finally, Sect 5 concludes the paper with key findings and insights.

## 1 Related works and contributions

### 1.1 Related works

Currently, extensive research has been conducted on UAV-related channel modeling and characterization. From a perspective of modeling methodologies, research can generally be classified into two approaches: deterministic and stochastic models [10]. Deterministic models aim to precisely predict the behavior of radio propagation based on specific physical parameters and environmental information. Ray tracing stands out as a prominent and representative technique, which models radio propagation by representing signals as rays. For instance, the authors in [7] investigated A2G channel models for low-altitude UAVs and validated ray-tracing simulation results against actual measurements in a suburban environment. Furthermore, a series of measurement campaigns in urban settings analyzed the delay and received power of multipath components (MPCs), with ray-tracing tools employed to corroborate the measurement findings in [8].

Unlike deterministic models that rely on precise environmental details, stochastic models utilize randomness and probability distributions to characterize channel properties such as angular distribution and multipath effects. Among those stochastic models, the geometry-based stochastic model (GBSM) is widely adopted due to its low computational complexity and adaptability to diverse scenarios. Extensive research has been conducted on GBSMs, including typical works like [10–12]. In [13], Ma et al. proposed a three-dimensional (3-D) non-stationary GBSM for A2A communication channels, incorporating a 3-D Markov mobility model to simulate UAV movements in both horizontal and vertical directions. In [14], Zeng et al. studied the UAV A2A communication channel based on two-cylinder reference model. This model considers the LoS, single-bounced (SB), and double-bounced (DB) rays. Furthermore, Hu et al. used UAVs as relay base stations for low Earth orbit (LEO) satellite communications, establishing a space-air-ground integrated communication link [15]. They proposed a 3-D multiple-input and multiple-output (MIMO) channel model based on regular geometry and analyzed key parameters, including the space-time correlation function, Doppler power spectral density (DPSD), level crossing rate (LCR), and average fading duration (AFD). However, GBSMs often exhibit limited simulation accuracy in specific scenarios, besides, most studies focus on the derivation of channel impulse response (CIR) and small-scale fading characteristics, the effects of trees on radio propagation are rarely considered.

Regarding trees’ impact on radio signals, Torrico et al. studied the path loss in a vegetated residential environment at 900 MHz, modeling trees as an absorbing phase screen [16]. The authors in [17] proposed an empirical path loss model for forest environments across multiple frequencies, including 870 MHz and 2.4 GHz. Genc developed a path loss model for pine forests, considering frequency, distance, and volumetric occupancy at two sub-6G frequency bands, 3.5 GHz and 4.2 GHz [18]. These studies primarily address large-scale fading characteristics and do not cover mmWave frequency bands. Lv et al. conducted mmWave dual-polarized propagation channel measurements at 39 GHz to investigate the impact of foliage between transceivers [19]. The results show that vegetation introduces significant channel dispersion in the delay domain compared to a pure LoS scenario. In [20], Picallo studied the device-to-device communication links blocked by oak and pine trees at 2.4 GHz. The mass of oak tree leaves is modeled as a cube, and pine tree is abstracted as a cone-shaped structure with superposition of greater to less length layers starting from the branches closer to the ground. However, this model fails to consider the fractal structural characteristics of the tree. While these references provide valuable insights into the effects of trees on radio propagation, there remains a gap in research specifically addressing UAV A2G channel characteristics in vegetation scenarios.

### 1.2 Contributions

In this paper, we explore the wireless channel characteristics at 28 GHz in vegetation scenarios. The key contributions of this paper are listed as follow.

1. We utilize fractal theory to model 3-D trees, considering factors including recursion depth, branching angle, and branching ratio. Besides, the Weibull distribution is used to depict trees’ size, and the uniform distribution is used to model their spatial deployment.
2. The stochastic propagation-graph channel model is employed to simulate the UAV A2G wireless communication link in vegetation scenario. This model considers the impact of trees and the ground, incorporating a maximal propagation delay constraint to select effective points. For the non-LoS (NLoS) components originating from the ground, a directive scattering model is used to enhance modeling accuracy.
3. Using the proposed method, we analyze the UAV A2G wireless channel characteristics. Specifically, we investigate the spatial CCF, time-varying power delay profile (PDP), DPSD, and power angular profile (PAP). Additionally, the effects of varying UAV hovering heights and radii on the delay spread are also studied.
4. It is noteworthy that our verification is based solely on computer simulations and currently lacks experimental validation. Instead, the proposed channel modeling framework advances methodological and analytical approaches for forest environments, in line with current established channel modeling methodologies.

## 2 Non-stationary stochastic propagation-graph channel model

**Fig 1:**
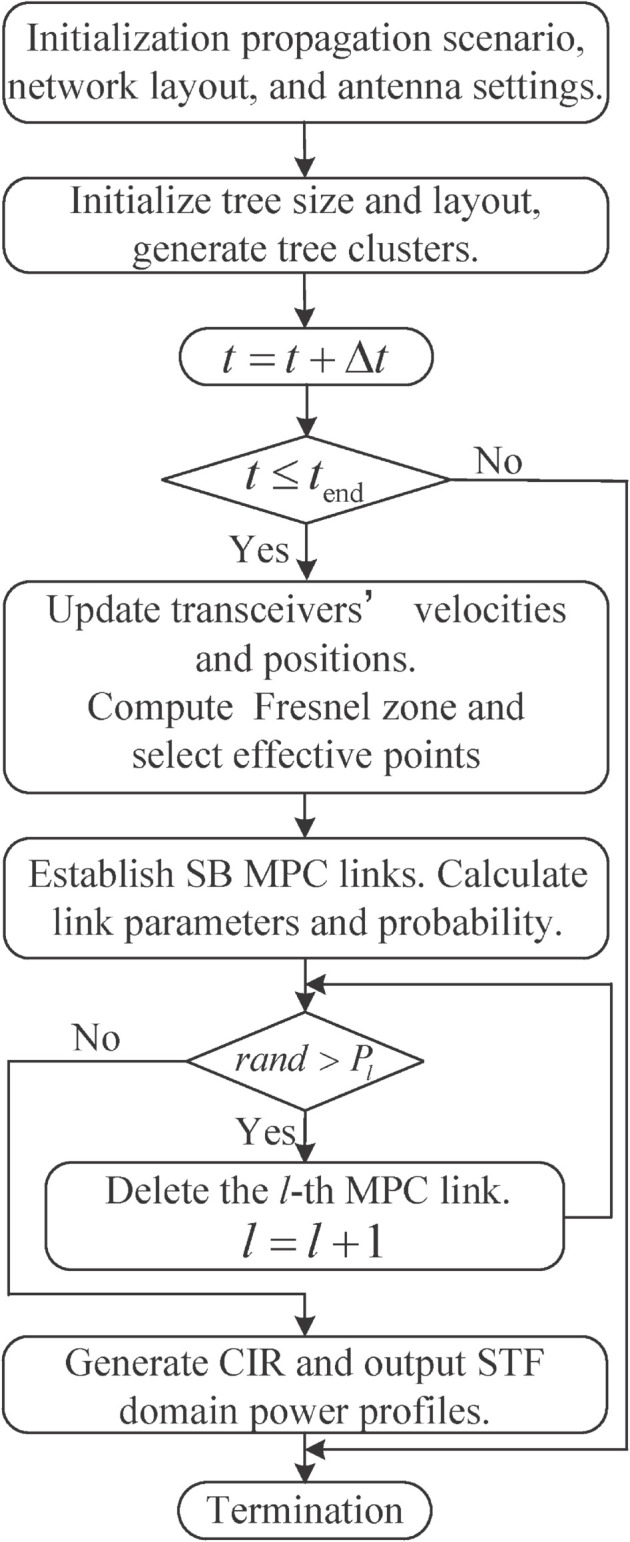
The flowchart of the proposed channel modeling procedure.

The proposed channel modeling algorithm in presented in Fig 1 and the major steps are given as follows.

1. Initialize the simulation environment, including the propagation scenario, network layout, and antenna settings. Generate the ground cluster and tree clusters with random size and positions, abstract them into vertices.
2. Update transceivers’ velocities and positions. Compute the Fresnel zone and select the effective points located within this region. Establish SB MPC links and assign a survival probability to each MPC link based on the position relationship. Delete MPC links according to their survival probability.
3. Compute the CTF of each existing MPC link. Generate the CIR based on the propagation-graph theory and output the STF domain power profiles. Repeat Step 2 and Step 3 until the termination conditions are met.

### 2.1 Modelling and initialization of trees

#### 2.1.1 3-D fractal tree model.

Current findings demonstrate that fractal-based approaches excel at replicating botanical structures with remarkable fidelity. A fractal tree model is a computational framework that mimics the branching patterns of trees and plants using fractal geometry. It is based on the principle of self-similarity, where smaller branches recursively replicate the structure of larger ones at different scales.

A fractal tree is a mathematical and graphical representation of a tree-like structure with fractal properties. It consists of self-similar structures at various scales and is typically created using recursive algorithms [21]. To construct a fractal tree, begin with a trunk of any length. Generate several branches from the trunk, each slightly smaller and oriented in various 3-D directions. For each of these branches, generate additional branches, applying similar scaling and rotation. This process is repeated iteratively to build the tree’s structure.

**Fig 2:**
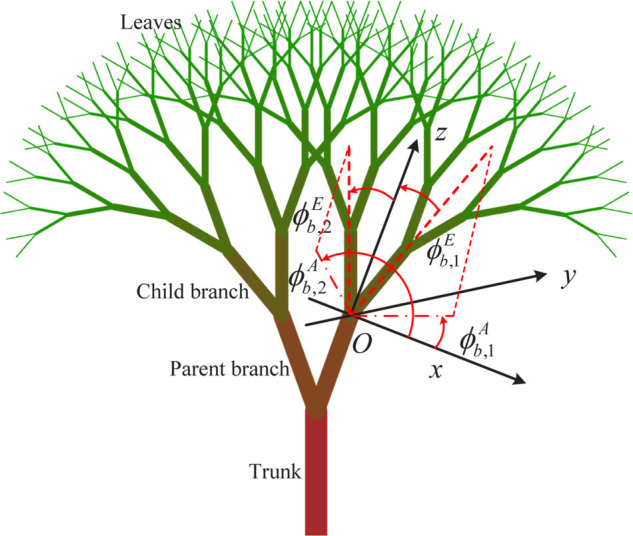
A diagram of the geometric fractal tree.

As introduced above, a 3-D fractal tree exhibits self-similarity and recursive branching patterns and can be controlled by these key parameters as shown in Fig 2.

1. Recursion depth *N*_*r*_. The number of iterations or levels of recursion applied to generate the tree. Greater depth results in a more complex and detailed tree with more branches. Shallow recursion creates a simpler tree.
2. Number of branches *N*_*b*_. The number of branches that split off from each parent branch at each iteration. More branches create a fuller and denser tree. Fewer branches result in a sparser tree.
3. Branching angle $\phi_{b}^{E}$. It refers to the angle at which a branch splits from its parent branch. Take the parent branch as the *z*-axis, then it means the elevation angle. Generally, a smaller angle results in branches that are closer together and can create a denser, more compact tree. A larger angle results in branches that spread out more, leading to a more open and wide-spreading tree.
4. Branch splitting angle $\phi_{b}^{A}$. It involves the splitting branch angles in the fractal generator that are calculated. It can be regarded as the azimuth angle in contrast to the branching angle $\phi_{b}^{E}$.
5. Branching ratio $\gamma_{b}$. The ratio of the length of a child branch to its parent branch. This parameter controls how quickly branches shorten as you move up the tree. A small ratio results in short, stubby branches, while a large ratio results in longer branches.

The key parameters of a fractal tree determine its shape, structure, and overall appearance. According to reference [22], the position affine transformations between parent branch $p_{n-1}$ and child branch $p_{n}$ can be calculated as

$$p_{n}=p_{n-1}+r\underset{{𝐑}_{A}}{\underbrace{\left[\begin{array}{ccc} \cos\phi_{b}^{A} & -\sin\phi_{b}^{A} & 0\\ \sin\phi_{b}^{A} & \cos\phi_{b}^{A} & 0\\ 0 & 0 & 1 \end{array}\right]}}\underset{{𝐑}_{E}}{\underbrace{\left[\begin{array}{ccc} 1 & 0 & 0\\ 0 & \cos\phi_{b}^{E} & -\sin\phi_{b}^{E}\\ 0 & \sin\phi_{b}^{E} & \cos\phi_{b}^{E} \end{array}\right]}}\gamma_{b},$$ (1)

where *r* is the branch length, which is set to a constant value in this paper. ${𝐑}_{A}$ and ${𝐑}_{E}$ are the two rotation matrices related to the azimuth angle $\phi_{b}^{A}$ and elevation angle $\phi_{b}^{E}$ for each iteration, respectively. $p_{n}$, $p_{n-1}$, and $\gamma_{b}$ are column vectors. By tuning these parameters approximately, it can mimic real trees more closely, from highly symmetrical and structured trees to irregular and natural-looking ones.

#### 2.1.2 Distribution of trees.

Within a given geographical area, trees are randomly distributed according to a density ratio parameter that determines the average number of trees per unit area. As such, the radio signal may interact with multiple trees along the transmission path in practical radio propagation scenarios. Herein, the spatial distribution is assumed to follow the random uniform distribution in two-dimensional (2-D) space.

$$f(x,y)=\left\{ \begin{array}{cc} \frac{1}{\text{Area}(D)} & \text{If }(x,y)\in D,\\ 0 & \text{Otherwise}, \end{array}\right.$$ (2)

where *D* denotes the 2-D distribution region, and Area(·) returns the area value.

The size distribution of these trees follows a Weibull probability density function [23,24], with an expression of

$$f(u;a,b)=\{\begin{array}{c} \frac{b}{a}{\left(\frac{u}{a}\right)}^{b-1}e^{-{\left(\frac{u}{a}\right)}^{b}},u\geq 0\\ 0,u<0. \end{array}$$ (3)

where *a* (scale parameter) and *b* (shape parameter) characterize the distribution, with mean tree size given by 𝔼(u)=aΓ(1  +  1/b). For each generated tree with initial dimensions R=L×W×H, the Weibull random process transforms its size to $R^{\prime }=u\left(L\times W\times H\right)$, effectively scaling all three dimensions by the random variate *u* sampled from the Weibull distribution while preserving the original aspect ratio.

### 2.2 Selection of effective points

When modeling, it is crucial to first establish a digital map based on the layout of transceivers and other obstacles. Considering all vertices in a large simulation area can impose a significant computational burden. Therefore, our goal is to create a smaller, more manageable vertex set with fewer but effective points. To achieve this, we use maximal propagation delay constraint criteria, which assumes that most of the signal power is concentrated within a few delay taps. The details of this approach are outlined as follows.

1. *Tree points generation.* In the vegetation scenario, the scattering effects of trees on radio propagation are significant. However, the irregular surfaces of trees make it challenging to discretize them into points. To address this, we use the fractal tree generation method to represent the trees. Given the computational complexity associated with a large number of tree points, the generated fractal tree vertices are downsampled to 100 points per tree, as it suffices to capture the essential tree structure and the scattering characteristics while substantially reducing computational overhead.
2. *Ground points generation.* In the A2G communication scenario, the ground plays a crucial role. We consider scatterers around the single-bounce reflection point (RP), which can be easily determined based on geometric relationships. Initially, we discretize the ground plane into points placed evenly, with the distance between any two points set to *δ* to satisfy the far-field condition, as follows
  $$2\delta^{2}/\lambda \leq \min\{r_{1},r_{2}\},$$ (4)
  where *r*_1_ and *r*_2_ denote the link distances of transmitter (Tx)-RP and receiver (Rx)-RP, respectively.
3. *Selection of effective points.* Usually, obstacles located in the neighbor exert predominant effects on the received power, and MPCs stemming from far obstacles may be overwhelmed by the thermal noise. Herein, we consider the delay $\tau_{\text{max}}$-constraint Fresnel ellipsoid zone, which is expressed as
  $$|r_{T,s}+r_{s,R}|-r_{TR}\leq c_{0}\tau_{\text{max}},$$ (5)
  where *r*_*T*,*s*_ is the distance from Tx to the point *s*, *r*_*s*,*R*_ means the distance from the point *s* to Rx, *r*_*TR*_ is the LoS link distance, and *c*_0_ is the speed of light. Based on this criteria, all discrete points are filtered, and the remaining are considered as effective points in the following modeling procedure. Using these effective points within the Fresnel ellipsoid zone, we establish single-bounce NLoS links. Note that an NLoS link vanishes if the corresponding effective point moves outside the zone as the transceiver moves. Similarly, new NLoS links may emerge when the zone encompasses new effective points, reflecting the so-called birth–death phenomenon.

### 2.3 Channel model

#### 2.3.1 Propagation graph channel model.

The propagation-graph channel modeling theory was first proposed by Pedersen T. and Fleury B. H. in 2006 at the Vienna Mathematical Modeling Forum [25]. This theory abstracts the wireless radio wave propagation environment into a graph, where the discrete abstract vertex is used to represent antennas and the possible scattering objects, and the directed edges are utilized to describe the possible propagation path of radio waves. On this basis, it can emulate those physical phenomena such as reflection and scattering propagation experienced by radio waves. Finally, it uses the cascade of frequency domain transfer functions to obtain the final channel transfer function (CTF) considering multiple-bounce propagation.

**Fig 3:**
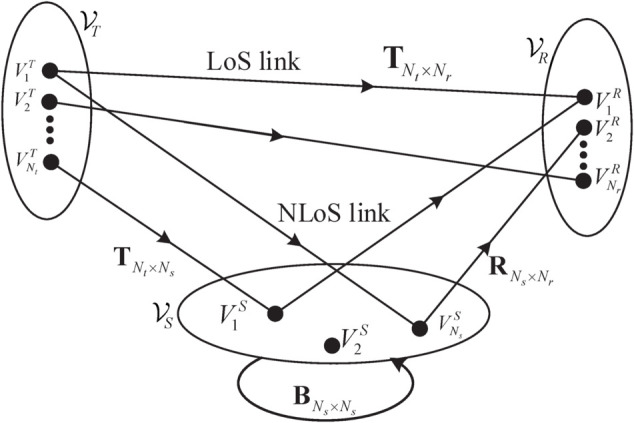
A diagram of the propagation graph channel model.

Fig 3 presents a diagram of the propagation-graph model. As reported in [25], an abstract graph consists of edge sets ℰ and vertex sets 𝒱. Specifically, 𝒱 contains the Tx vertex set ${𝒱}_{T}=\{V_{1}^{T},V_{2}^{T},\cdots ,V_{N_{t}}^{T}\}$, the Rx vertex set ${𝒱}_{R}=\{V_{1}^{R},V_{2}^{R},\cdots ,V_{N_{R}}^{R}\}$, and the scatterer vertex set ${𝒱}_{S}=\{V_{1}^{S},V_{2}^{S},\cdots ,V_{N_{s}}^{S}\}$. Note that *N*_*t*_, *N*_*r*_, and *N*_*s*_ represent the element numbers of these 3 different sets, respectively. Then, the vertex set can be expressed as $𝒱={𝒱}_{T}\cup {𝒱}_{R}\cup {𝒱}_{S}$. ℰ denotes the abstracted edges among different vertices, which contain the following 4 types, i.e., edges from Tx to Rx ${ℰ}_{D}=\{e_{ij}|e_{ij}\in {𝒱}_{T}$ × ${𝒱}_{R}\}$, edges from Tx to scatterers ${ℰ}_{T}=\{e_{ij}|e_{ij}\in {𝒱}_{T}$ × ${𝒱}_{S}\}$, edges from scatterers to Rx ${ℰ}_{R}=\{e_{ij}|e_{ij}\in {𝒱}_{S}$ × ${𝒱}_{R}\}$, and edges from scatterers to scatterers ${ℰ}_{S}=\{e_{ij}|e_{ij}\in {𝒱}_{S}\times {𝒱}_{S}\}$. Based on these 4 different types of propagation edges, the propagation-graph model can emulate the LoS and NLoS links by cascading different edges. For example, a SB NLoS link can be decomposed as two edges of ${𝒱}_{T}$-${𝒱}_{S}$ and ${𝒱}_{S}$-${𝒱}_{R}$.

For a given edge *e*, the corresponding frequency-domain CTF is presented as

$$A_{e}(f)=g_{e}\exp\{-j(2\pi \tau_{e}f+\phi_{e})\},$$ (6)

where *g*_*e*_ denotes the edge gain, $\tau_{e}$ is the corresponding propagation delay with an expression of $\tau_{e}=D_{e}/c_{0}$, *D*_*e*_ is the edge distance, and *c*_0_ is the speed of light. $\phi_{e}$ is a phase variable, which is randomly distributed within a range of [0,2π). *f* is the frequency domain variable.

#### 2.3.2 Edge gain.

**Fig 4:**
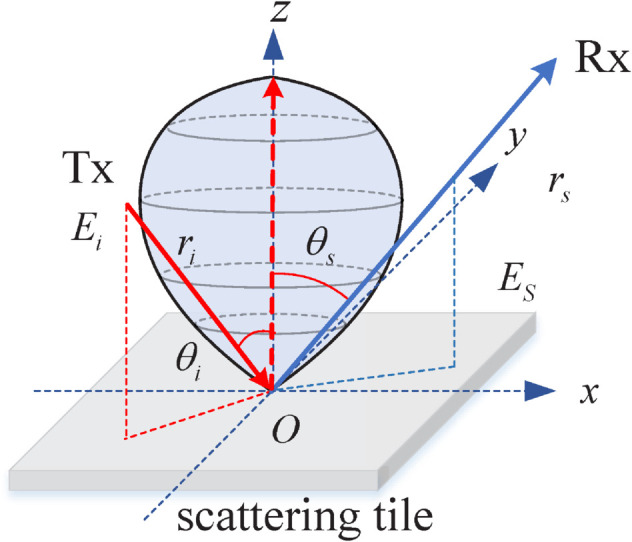
A diagram of Lambertian scattering model.

1. *Trees*. Obviously, an accurate link gain can provide a good depiction of the propagation environment. Herein, the propagation graph model is used to characterize the radio waves via trees. For these aforementioned 4 types of edges, the corresponding gain expressions based on [26] are expressed as
  $$g_{e}^{2}=\left\{ \begin{array}{cc} {\left(\frac{1}{4\pi f\tau_{e}}\right)}^{2}, & e\in {ℰ}_{D}\\ \frac{1}{4\pi f\mu \left({ℰ}_{T}\right)}\cdot \frac{\tau_{e}^{-2}}{S\left({ℰ}_{T}\right)}, & e\in {ℰ}_{T}\\ \frac{1}{4\pi f\mu \left({ℰ}_{R}\right)}\cdot \frac{\tau_{e}^{-2}}{S\left({ℰ}_{R}\right)}, & e\in {ℰ}_{R}\\ \frac{g^{2}}{\text{odi}i(e)}, & e\in {ℰ}_{S} \end{array}\right.,$$ (7)
  where g∈(0,1) is a constant attenuation factor caused by scattering or reflection. The first case is derived based on the free space propagation. The last case refer to the inner-propagation among scatters. As for the middle two cases related to ${ℰ}_{T}$ and ${ℰ}_{R}$, edges are normalized to have average gain following a law of inversely proportional to the the distance, hence, function μ(·) calculates the average propagation delay and it can be expressed as
  $$\mu (ℰ)=\frac{\sum_{e\in ℰ}\tau_{e}}{\text{card}\{ℰ\}},$$ (8)
  where function card(·) returns the element number of a given set ℰ. The function S(·) provides a normalized factor with an expression of
  $$S(ℰ)=\sum_{e\in ℰ}\tau_{e}^{-2}.$$ (9)
  Function odi(·) in Eq (7) calculates the number of edges that are connected to this vertex. Regarding the single-bounce NLoS links introduced by trees, these primarily involve edges in ${ℰ}_{T}$ and ${ℰ}_{R}$. Although the propagation graph model employs a gain factor *g* to adjust link gains—which somewhat lacks physical interpretation—this approach remains well-suited for tree scattering due to the computational complexity of accurately modeling wave interaction with trees, given their intricate structures involving leaves and branches oriented in various directions.
  Another important consideration is the omission of tree-tree radio wave interactions. This simplification is justified for two main reasons. First, the majority of the received power is contributed by the LoS and single-bounce NLoS components; hence, double- or higher-order bounces involving multiple trees have only a marginal effect on the final signal. Second, computing such tree-tree transmission components is highly time-consuming, as the associated computational complexity scales as $𝒪(N_{S}^{2})$.
2. *Ground*. From Eq (7), we can learn that each edge gain is totally independent of another even for the same link. Hence, it lacks some physical meanings to some degree. To address this issue, Tian et al. proposed a semi-deterministic channel model, which elaborates the scattering components by using the Lambertian scattering model [27]. This model has been widely validated on a rough impinging surface based on some experiments. As shown in Fig 4, radio waves impinging on the soil ground undergo scattering that approximately follows the Lambertian model. This model dictates that the peak scattering power is always in the direction perpendicular to the surface, regardless of the incident angle. Compared to the widely used GBSMs, this model can obtain high simulation accuracy verified by actual measurements. In this paper, we utilize this model to emulate the scattering effects via the soil ground. Considering the single-bounce via the ground, the Lambertian-based link gain in [27] can be expressed as
  $$g_{e}^{2}=\left\{ \begin{array}{cc} \frac{\Delta S\cdot \cos\left(\theta_{i}\right)}{4\pi r_{i}^{2}}, & e\in {ℰ}_{T}\\ \frac{S^{2}\cos\left(\theta_{s}\right)}{\pi r_{s}^{2}}\cdot \frac{\lambda^{2}}{4\pi }, & e\in {ℰ}_{R} \end{array}\right.,$$ (10)
  where *r*_*i*_ is the link distance from Tx to point belonging to ${ℰ}_{T}$, and *r*_*s*_ means the link distance from point to Rx belonging to ${ℰ}_{R}$. ΔS is the scattering tile area, S∈(0,1) represents the scattering loss factor, $\theta_{i}$ is the incident angle between the incident wave and the normal direction of the scattering surface, and $\theta_{s}$ is the scattering angle between the scattering wave and the normal direction of the scattering surface.

#### 2.3.3 Channel impulse response.

Regarding the MIMO communication system, the corresponding CTF at time *t* marked as H(t,f) with dimensions of $N_{T}\times N_{R}$, is the superposition of the LoS component D(t,f) with dimensions of *N*_*T*_ × *N*_*R*_ and the NLoS component, which can be obtained via the cascade CTF of edges ${ℰ}_{T}$ and ${ℰ}_{R}$. On the assumption of *K* trees located within the effective coverage range, we abstract each tree into an individual vertex set. Note that each tree cluster is treated as an individual subgraph, consisting of edges from the Tx to the *k*-th tree and from the *k*-th tree to the Rx. We consider only single-bounce paths via trees and ground, neglecting multiple reflections. Furthermore, multiple-bounce paths at mmWave frequencies involving trees are expected to be significantly weaker than single-bounce and LoS components due to high penetration loss and diffuse scattering from vegetation. Then, the single-bounce CTF is expressed as

$$H(t,f)=D(t,f)+\sum_{k=1}^{K}T_{k}(t,f)R_{k}(t,f)+T_{G}(t,f)R_{G}(t,f),$$ (11)

where $T_{k}(t,f)$ and $R_{k}(t,f)$ are the time-varying CTFs of edges ${𝒱}_{T}$-${𝒱}_{S,k}$ and ${𝒱}_{S,k}$-${𝒱}_{R}$, with dimensions of *N*_*T*_ × *N*_*k*_ and *N*_*k*_ × *N*_*R*_, respectively. In this model, $T_{k}$ denotes the transfer matrix from the Tx to the vertices of the *k*-th tree, while $R_{k}$ represents the transfer matrix from these vertices to the Rx. The symbol *N*_*k*_ indicates the cardinality (i.e., the number of vertices) of the *k*-th tree’s vertex set. Likewise, $T_{G}(t,f)$ and $R_{G}(t,f)$ are those transfer matrices via the ground. By applying the inverse Fourier transform, the corresponding time-varying CIR can be obtained, expressed as

$$h(p,q,t,\tau )={ℱ}^{-1}\{H(p,q,t,f)\}.$$ (12)

Note that *H*(*p*, *q*, *t*, *f*) is the element of MIMO matrix $H_{N_{T}\times N_{R}}$, where *p* and *q* indicate the antenna index of the *p*-th Tx and *q*-th Rx, *t* is the time variable, and *f* is the frequency variable, respectively.

## 3 Channel statistical properties

### 3.1 RMS DS

To quantify the dispersion in the time delay domain caused by multipaths, the root-mean-square (RMS) delay spread is commonly used. It is defined as

$$\sigma_{\tau }=\sqrt{\frac{\sum_{l}^{L}\tau_{l}^{2}P(\tau_{l})}{\sum_{l}^{L}P(\tau_{l})}-{\left(\frac{\sum_{l}^{L}\tau_{l}P(\tau_{l})}{\sum_{l}^{L}P(\tau_{l})}\right)}^{2}},$$ (13)

where *L* represents the total number of multipaths, $\tau_{l}$ denotes the propagation delay of the *l*-th multipath, and $P(\tau_{l})$ is the corresponding received power. Assuming the transmit power *P*_0_ = 1 W, we have $P(\tau_{l})=\|h(\tau_{l}){\|}^{2}$.

### 3.2 Spatial CCF

The spatial CCF characterizes the statistical relationship between signals received at different antenna elements. It quantifies how the fading channels at distinct spatial locations are correlated, which is crucial for designing the multiple-input multiple-output (MIMO) systems, beamforming, and diversity schemes. The spatial CCF is typically defined as

$$R(\Delta 𝐝)=𝔼\left[h(𝐫,t)\;h^{*}(𝐫+\Delta 𝐝,t)\right],$$ (14)

where h(𝐫,t) is the channel impulse response at position **r** and time *t*, Δ𝐝 is the spatial displacement vector between antennas, 𝔼[·] denotes the statistical expectation, and ${\left(\cdot \right)}^{*}$ represents complex conjugation.

### 3.3 Spatial-temporal-frequency power profiles

Spatial-Temporal-Frequency (STF) power profiles characterize the distribution of MPC power across spatial, temporal, and frequency domains in wireless communication channels. These profiles capture how multipath propagation, user mobility, and frequency-selective fading influence signal strength variations over different locations, time instants, and frequency bands. By analyzing these profiles, we can quantify key channel properties such as coherence time, bandwidth, and distance.

Considering a time-varying propagation scenario with *N*(*t*) effective vertexes at time *t*, the single-bounce assumption yields *N*(*t*) distinct propagation links whose contributions superimpose to form the received signal. Each *n*-th link originating from vertex *s*_*n*_ is characterized by the parameter set ${𝒫}_{n}=\{g_{n},\tau_{n},f_{n},\psi_{n}^{A/D},\theta_{n}^{A/D}\}$, where *g*_*n*_ represents the complex gain, $\tau_{n}$ denotes the propagation delay, *f*_*n*_ indicates the Doppler frequency shift, and $\psi_{n}^{A/D}$ and $\theta_{n}^{A/D}$ specify the azimuth and elevation angles of arrival/departure, respectively. The Doppler frequency shift *f*_*n*_ is determined by the relative motion through

$$f_{n}=\frac{1}{\lambda }\langle v_{\text{u}},\frac{d_{un}}{\left\|d_{un}\right\|}\rangle +\frac{1}{\lambda }\langle v_{\text{t}},\frac{d_{tn}}{\left\|d_{tn}\right\|}\rangle ,$$ (15)

where $v_{\text{u}}$ and $v_{\text{t}}$ are the speed vectors of flying UAV and ground terminal, respectively. $d_{un}$ and $d_{tn}$ refer to the links of terminal-*s*_*n*_ and UAV-*s*_*n*_, respectively. Operator ⟨·,·⟩ calculates the vector inner product, and ‖ ⋅ ‖ returns the vector norm. The angular variables for the *n*-th MPC requires the positions of transceiver and vertex, without loss of generality, the elevation and azimuth angles of link UAV-*s*_*n*_ can be expressed as

$$\theta_{un}=\arcsin\left(\frac{z_{n}-z_{u}}{\sqrt{(x_{n}-x_{u}{)}^{2}+(y_{n}-y_{u}{)}^{2}+(z_{n}-z_{u}{)}^{2}}}\right),$$ (16)

$$\psi_{un}=\{\begin{array}{c} \arccos\left(\frac{x_{n}-x_{u}}{\sqrt{(x_{n}-x_{u}{)}^{2}+(y_{n}-y_{u}{)}^{2}}}\right),\text{if}\;y_{n}\geq y_{u}\\ -\arccos\left(\frac{x_{n}-x_{u}}{\sqrt{(x_{n}-x_{u}{)}^{2}+(y_{n}-y_{u}{)}^{2}}}\right),\text{if}\;y_{n}<y_{u}. \end{array}$$ (17)

where $p_{u}=[x_{u},y_{u},z_{u}{]}^{⊤}$ and $p_{n}=[x_{n},y_{n},z_{n}{]}^{⊤}$ denote the positions of UAV and vertex *s*_*n*_, respectively.

To obtain the power profiles in STF domains, the signal reconstructed method is adopted, including the following main procedures:

1. Determine the sampling resolution Δr. For PDP, the delay resolution is related the bandwidth *B*, i.e., Δr=1/B. For DPSD, the Doppler shift resolution is inversely proportional to time window length *T*_*c*_, expressed as $\Delta r=1/T_{c}$. Regarding the PAS, it is related to the antenna array aperture *A*, with respects to Δr=1/A.
2. Determine the sampling range $v_{\text{min}}$ and $v_{\text{max}}$. The minimal and maximal values can be obtained by iterating all links.
3. Sampling and reconstruction. Reconstruct STF power profiles based on the sampling results, expressed as
  $$S(t,v)=\sum_{n=0}^{N(t)}g_{n}^{2}\cdot \frac{\sin\left[\pi (v-v_{n})/\Delta r\right]}{\pi (v-v_{n})/\Delta r}\cdot \underset{A}{\underbrace{\frac{\cos(\beta \pi (v-v_{n})/\Delta r)}{1-(2\beta (v-v_{n})/\Delta r{)}^{2}}}},$$ (18)
  where variable *v* can be replaced by the delay *τ*, Doppler shift *f*, elevation angle *θ*, and azimuth angle *ψ*. Part *A* represents the cosine roll-off characteristics [28]. Limited by the range, the discrete values of variable *v* is expressed as
  $$v=v_{\text{min}}+k\Delta r,k=0,1,\cdots ,(v_{\text{max}}-v_{\text{min}})/\Delta r.$$ (19)
  $v_{n}$ denotes the corresponding parameter value of the *n*-th link. *n* = 0 is the case of LoS link, and *β* is the roll-off factor.

## 4 Simulation and analysis

### 4.1 Modeling of trees

**Fig 5:**
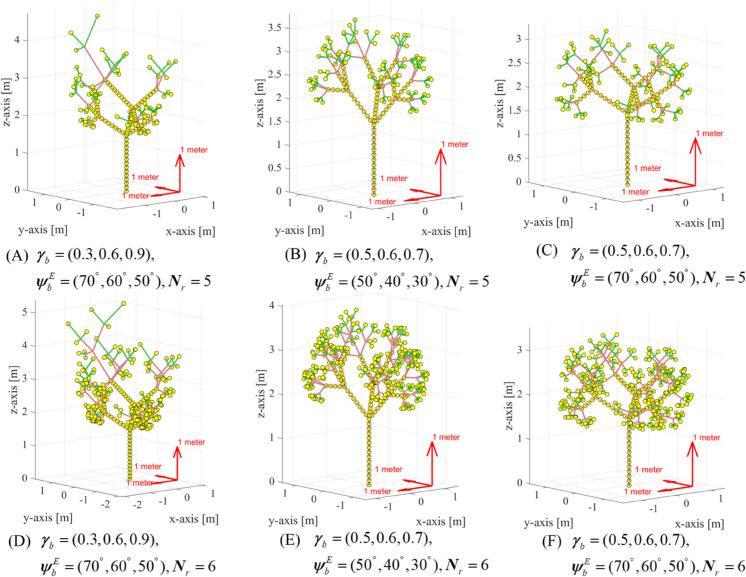
The 3-D diagram of fractal trees over different parameters, where $N_{b}=3$ and $\psi_{b^{A}}=(0^{\circ },120^{\circ },240^{\circ })$.

Fig 5 presents the 3-D geometric tree models generated under different parameters using the proposed method. Specifically, we examine the effects of tree recursion depth *N*_*r*_, branching angle $\phi_{b}^{E}$, and branching ratio $\gamma_{b}$, while keeping the number of branches fixed at *N*_*b*_ = 3 and the branch splitting angles constant at $\psi_{b}^{A}=(0^{\circ },120^{\circ },240^{\circ })$. Three key conclusions can be drawn from the results. (1) The recursion depth significantly influences the tree’s complexity; a greater recursion depth results in a more intricate tree with additional branches, which represents an older and more mature tree. (2) Regarding the branching ratio, parameters with low variance and a concentrated distribution lead to trees with densely distributed points, which more easily obstructs the LoS link. (3) For the branching angle, the differences on trees are minimal.

### 4.2 Simulation scenario layout

**Fig 6:**
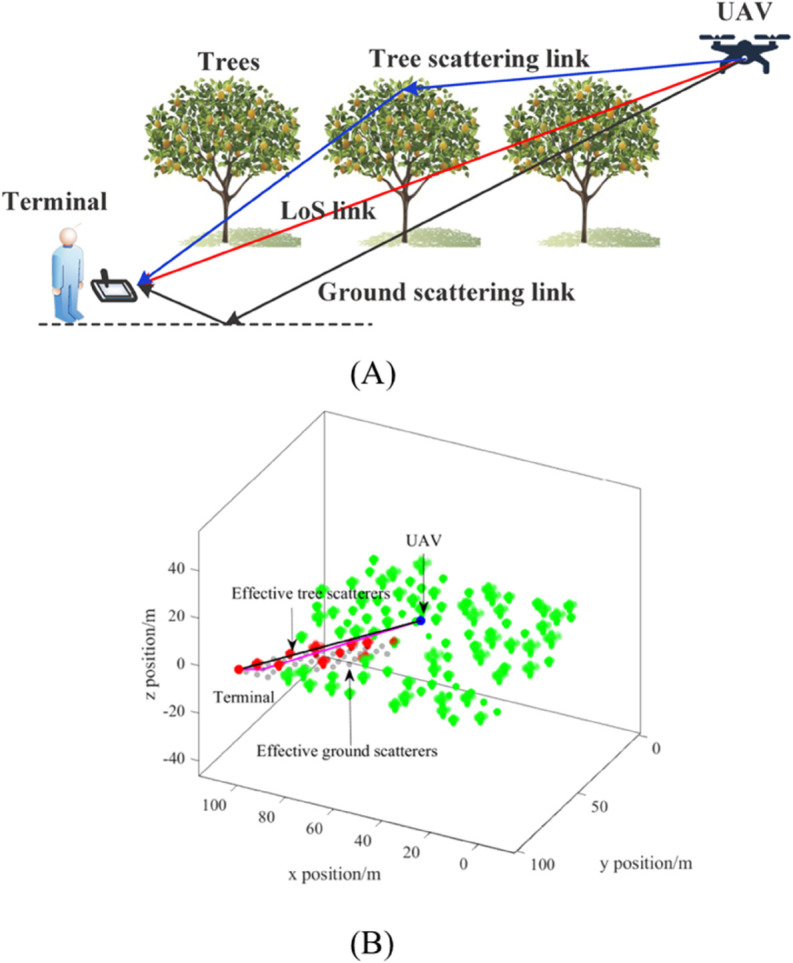
UAV A2G channel simulation scenario. (A) A diagram of the simulation scenario. (B) The established digital map.

Fig 6a illustrates the A2G wireless communication setup between a flying UAV and an on-ground terminal. Three distinct types of wireless links are identified, i.e., the LoS link, the ground-stemmed MPC link, and the tree-stemmed MPC link. 100 trees with different sizes are distributed randomly within a regular rectangular area with a size of 100m×100m. Fig 6b shows the corresponding digital map featuring trees, the UAV, the terminal, and the ground. Trees are represented by clusters of green points, with red points indicating effective tree points within the maximal propagation delay zone, and black points representing effective ground points. The UAV is depicted hovering above the trees and communicating with the user terminal via the wireless link. Specifically, the UAV performs a uniform circular motion with a radius of 20 meters at a constant speed of 30 km/h around the center of (50,50)m. Additional details of the simulation parameters are provided in Table 1.

**Table 1:** Simulation parameter settings.

Parameters	Values	Parameters	Values
Frequency carrier	28 GHz	Bandwidth	1 GHz
UAV speed	30 km/h	UAV height	20 m
Terminal location	(0,0,1.5) m	S factor	0.6
Updation interval	0.01 s	Simulation time	15.08 s
Hover center	(50,50) m	Maximal delay	9 ns
Scale parameter	1	Shape parameter	2
Number of branches *N*_*b*_	3	Branch splitting angle $\psi_{b}^{A}$	$\left(0^{\circ },120^{\circ },240^{\circ }\right)$
Branching ratio $\gamma_{b}$	(0.3,0.6,0.9)	Branching angle $\phi_{b}^{E}$	$\left(50^{\circ },40^{\circ },30^{\circ }\right)$
	(0.5,0.6,0.7)		$\left(70^{\circ },60^{\circ },50^{\circ }\right)$
Recursion depth *N*_*r*_	5,6	Hover radius	10m, 20m
Tx antenna number *N*_*T*_	1	Rx antenna number *N*_*R*_	1

### 4.3 PDP, delay spread, and spatial CCF

**Fig 7:**
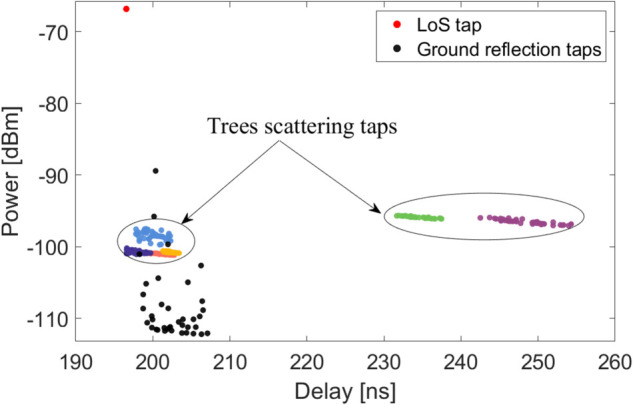
The PDP results at time 0s.

Based on the proposed modeling procedure, the time-varying virtual CIRs can be generated via the aforementioned parameters. Fig 7 plots a realization of the discrete PDP at the initialization time, i.e., the UAV position is (70,50,10)m. The LoS path with the highest received power is marked by a red solid circle. Besides, 7 SB-based tree clusters are generated and distinguished by solid circles with different colors. The solid black points refer to the paths coming from the ground. Obviously, the power of the LoS ray is over 25 dB higher than that of the SB NLoS paths. Regarding the time delay, the LoS tap corresponds to a value of 196.5ns, whereas the ground results in a range from 198.7ns to 207.1ns. The 7 trees result in relatively wide delay range, with a range from 196.5ns to 254.3ns. Moreover, we can learn that intra-cluster subpaths present a descending trend with a constant power decay rate regardless of different tree clusters. Besides, the distribution of intra-cluster subpaths stemming from ground component is relatively dense and irregular. A prominent subpath with higher power can be observed, which comes from the reflection mechanism, whereas the power of rest subpaths, stemming from scattering mechanism, is relatively lower. Note that the 7 clusters are identified based on the maximal delay constraint proposed in Sect 2.2.

**Fig 8:**
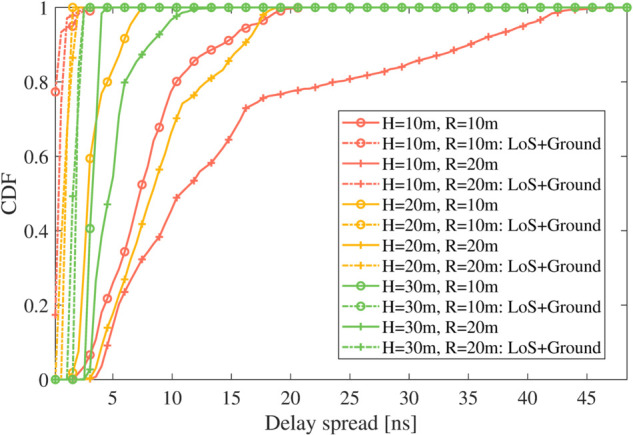
The CDF of delay spread comparison results between different UAV heights.

Fig 8 illustrates the cumulative distribution function (CDF) results for the entire simulation process, highlighting the influence of varying UAV heights and circular motion radii on delay spread. The UAV’s positions are centered at the same location, with heights of 10 m, 20 m, and 30 m, and radii of 10 m and 20 m, respectively. Additionally, we compare the CDFs of the delay spread resulting from the LoS and ground-stemmed components. Three significant insights emerge from the analysis. (1) An increase in the UAV’s hovering height leads to a corresponding increase in the delay spread, indicating a more dispersed signal path. (2) At lower UAV heights, the delay spread is comparatively smaller for the 10 m radius than for the 20 m radius. Interestingly, as the UAV’s height increases, this relationship shifts, and the delay spread becomes more pronounced for the smaller radius. (3) The inclusion of tree scattering introduces extensive NLoS components with large delays, whereas ground scattering produce NLoS taps with delays close to that of the LoS path due to the low height of the ground terminal. Consequently, the delay spread CDF for scenarios involving trees is significantly higher than that without trees, reflecting the broader distribution of delays introduced by vegetative scattering. These observations underscore the complex interplay between altitude and radius in determining signal behavior and can guide optimal UAV deployment for communication systems.

**Fig 9:**
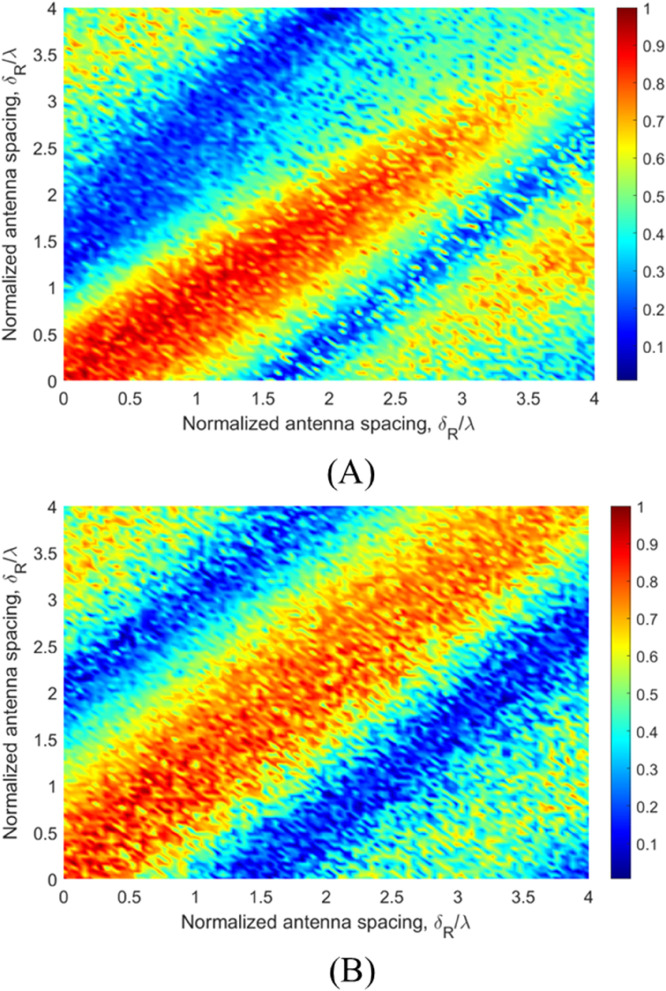
The spatial CCF results at the ground terminal. (A) Time *t* = 0 s. (B) Time *t* = 3.95 s.

Fig 9 presents the CCF results for the ground terminal at two distinct time instants: the initial UAV position (*t* = 0 s) and after completing a quarter circular motion (*t* = 3.95 s). The analysis explores both *x*- and *y*-axis directions across a 4-wavelength range. Key observations reveal that the spatial CCF matrix exhibits slow variation along its diagonal direction, which approximately aligns with the transmitter-receiver (Tx-Rx) link direction. Conversely, rapid correlation variations occur in the direction orthogonal to the Tx-Rx link, suggesting enhanced MIMO channel capacity potential in this orientation. When compared with the GBSM results at 28 GHz in a court yard scenario that may include some vegetation [28], our proposed model demonstrates distinct characteristics. The GBSM’s correlation coefficient decays to approximately 0.2 at just 2-wavelength separation, showing more rapid decorrelation than our results. This discrepancy stems from fundamental modeling differences: while the GBSM assumes uncorrelated scattering components, our propagation-graph-based approach explicitly incorporates the physical wave propagation mechanisms including LoS dominance, specular reflections, and diffuse scattering components. The preserved correlation structure in our model more accurately reflects realistic propagation environments where certain multipath components maintain spatial coherence over larger distances. Notably, we acknowledge that direct measurement-based evidence specifically for vegetation-influenced mmWave channels remains limited in the literature. To our knowledge, it still remains a gap that conducting extensive empirical spatial CCF measurements and analyses focusing explicitly on vegetated environments at mmWave bands. This represents a valuable direction for future experimental validation.

### 4.4 Time-varying spatial-temporal-frequency power profiles

**Fig 10:**
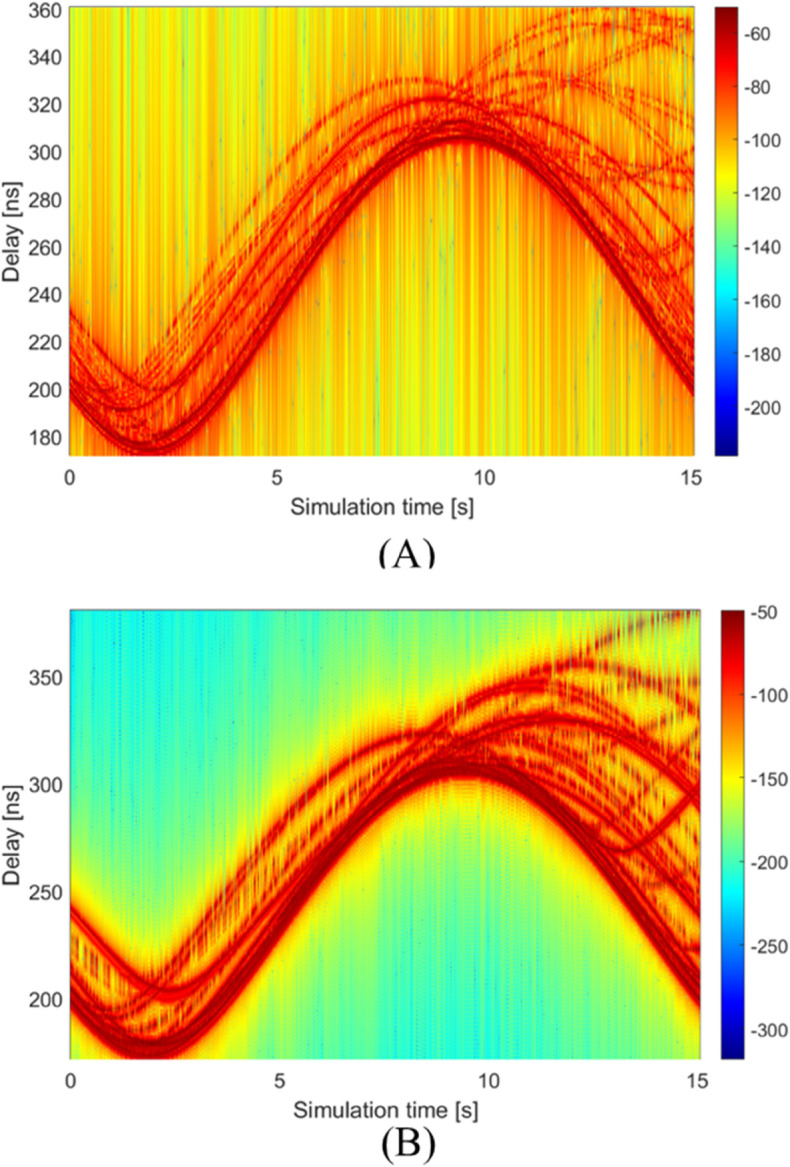
The time-varying PDPs. (A) PDPs without window filter. (B) PDPs with window filter.

Fig 10 presents the virtual PDP results obtained through our proposed method. Specifically, Fig 10a demonstrates the temporal evolution of PDP over the entire simulation duration, while Fig 10b compares the filtered PDP results using a cosine roll-off window based on Eq (18). Parameter *β* is set to 0.5 during the simulation. Three key observations emerge from these two figures. (1) The LoS component with the highest power exhibits a sinusoidal variation pattern, with its propagation delay calculated as $\tau_{\text{LoS}}=\|D_{0}$  +  R‖/c, where $D_{0}$ denotes the position vector from the terminal to the hovering center, and $R=R[\cos\omega t,\sin\omega t,0{]}^{\text{T}}$ represents the UAV’s trajectory vector. (2) The observed birth-death behavior of MPCs, characterized by their transient appearance and subsequent disappearance, manifests through two complementary physical mechanisms: varying Fresnel zones causing gradual, long-duration (>1s) MPC fluctuations, and intermittent tree blockages producing abrupt, short-duration (≤0.1s, corresponding to the channel updation interval) interruptions. These distinct temporal patterns provide conclusive evidence of the channel’s non-stationary characteristics in UAV communication scenarios. The Fresnel zone variations primarily affect signal propagation over extended periods, while tree blockages create rapid, localized disturbances. (3) The filtering analysis in Fig 10b reveals that while the window function effectively suppresses the noise floor by approximately 5 dB, it simultaneously introduces noticeable blurring in the MPC trajectories. This trade-off suggests that for accurate tracking of MPC temporal variations, the unfiltered PDP results provide superior clarity and are therefore recommended for trajectory analysis applications. It is caused by the fact that in our simulation scenario, which uses a large bandwidth of 1 GHz to achieve high delay resolution, the presence of multiple paths with small delay intervals makes the application of a window prone to causing multipath ambiguity.

**Fig 11:**
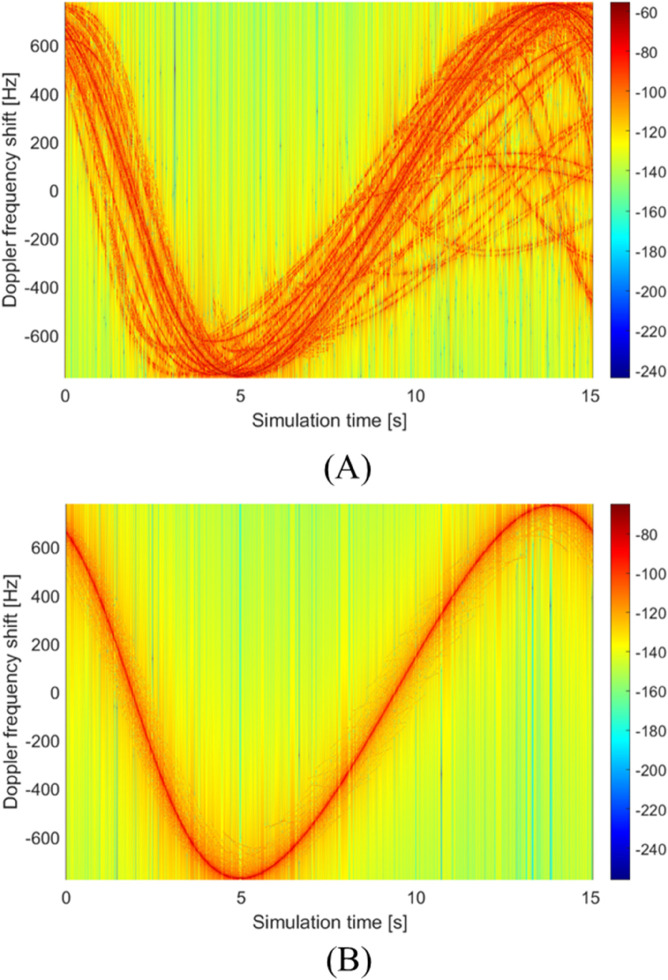
The time-varying DPSD results with different components. (A) LoS, trees, and ground-stemmed components. (B) LoS and ground-stemmed components.

The relative motion between transceivers and surrounding obstacles induces Doppler effects that contribute to channel non-stationarity, with the position-dependent DPSD results calculated using our proposed method shown in Fig 11. The time-varying DPSD spectra across different UAV positions reveal several key characteristics: MPC trajectories from LoS, ground reflections, and tree interactions exhibit distinct scattering patterns in Fig 11a, while Fig 11b specifically highlights how the LoS component forms a well-defined trajectory with approximately 15 dB higher power than the more scattered ground-reflected NLoS components distributed around it. The observed asymmetry in the LoS trajectory stems from time-varying angular geometry during UAV movement, demonstrating how motion dynamics affect signal characteristics, while surrounding trees generate clearly identifiable trajectories that further contribute to the complex Doppler spectrum. These combined observations of deterministic LoS behavior and stochastic multipath scattering provide crucial insights for modeling non-stationary channels in mobile scenarios, particularly highlighting the interplay between system mobility and environmental interactions in shaping the Doppler characteristics of UAV communication channels.

**Fig 12:**
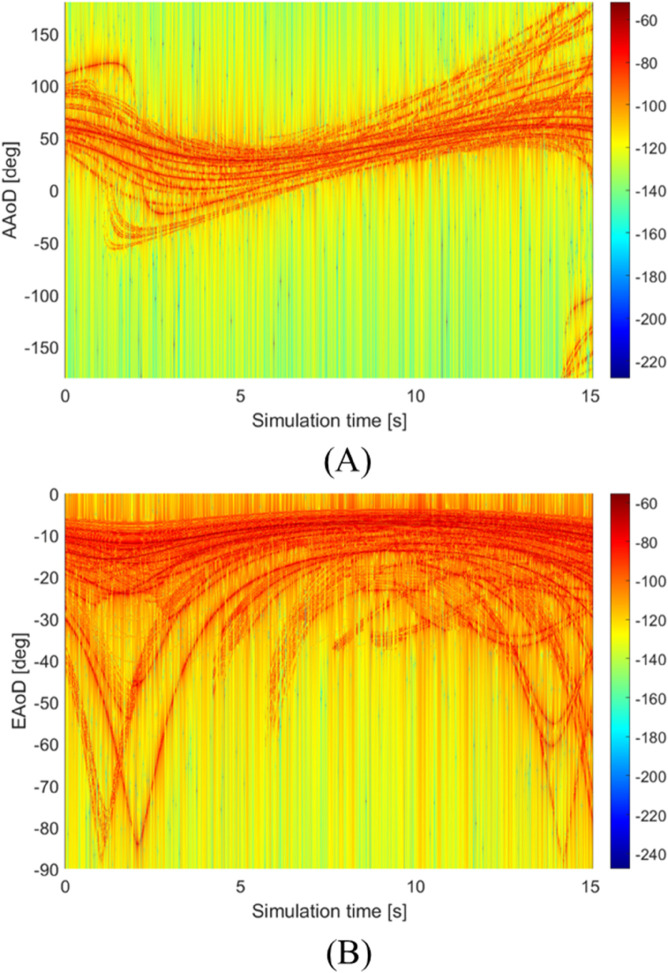
The time-varying PAP results. (A) PAP of AAoD. (B) PAP of EAoD.

Fig 12 presents the PAP results at various simulation times, with Fig 12a illustrating the azimuth outcomes and Fig 12b depicting the elevation results. A comparison between the figures reveals that the EAoD maintains a relatively narrow range, as shown in Fig 12b. This constrained variation in the PAP trajectory is attributed to the UAV’s predominantly horizontal movement. Conversely, the azimuth angle of departure (AAoD) spans a significantly wider range and shows a distribution pattern akin to that of the DPSD, where the MPC angles cluster around the LoS value. This highlights how UAV motion primarily influences azimuthal spreading while having a limited effect on elevation changes. Besides, the surrounding trees pose evident trajectories, especially in the PAP of AAoD.

## 5 Conclusions

In this paper, we investigate the modeling of wideband A2G wireless channels in the presence of trees at mmWave bands. First, the geometric fractal theory is employed to model trees. On this basis, we propose a novel UAV channel model based on stochastic propagation-graph theory and derive the virtual A2G CIR. We investigate the spatial CCF and effects of varying UAV heights and movement radii on delay spread. Furthermore, we comprehensively study the small-scale statistical properties in spatial-temporal-frequency domains, including the PDP, DPSD, and PAP. Simulation results demonstrate significant channel dispersion caused by trees and an increase in delay spread with higher UAV altitudes. The model exhibits clear non-stationary features (e.g. birth-death of MPCs) that align with realistic expectations. These results can inform the design and optimization of UAV communication systems.

Despite the insights provided, this study has certain limitations. The proposed model has currently been validated only through simulations; future work should include comparisons with empirical measurement data from real-world vegetated UAV channels to further assess its accuracy. Additionally, the model primarily focuses on single-bounce scattering; extending it to incorporate multi-bounce reflections could enhance its applicability in more complex environments. Other promising directions include considering more sophisticated UAV flight trajectories and refining the fractal vegetation model to account for seasonal variations in foliage.
